# Chemical profile, anti-biofilm and antioxidant activities of *Cymbopogon citratus* (DC.) Stapf essential oil

**DOI:** 10.1186/s12906-026-05431-1

**Published:** 2026-06-17

**Authors:** Asmaa M. Arafa, Galal Yahya, Mahmoud Saad Abdel-Halim, Ali Osman, Shaimaa I. Nazeih, Hisham A. Abbas, Islam Mostafa

**Affiliations:** 1 Department of Pharmacognosy, Faculty of Pharmacy, Zagazig University, Zagazig , 44519 Egypt; 2 Pharmaceutical Services Center, Faculty of Pharmacy, Zagazig University, Zagazig, 44519 Egypt; 3 Department of Microbiology and Immunology, Faculty of Pharmacy, Zagazig University, Zagazig, 44519 Egypt; 4 Department of Biochemistry, Faculty of Agriculture, Zagazig University, Zagazig, 44511 Egypt; 5 Clinical Pharmacy Program, Medical Sector, Zagazig National University, Tenth of Ramadan City, Egypt

**Keywords:** Volatile oil, Lemongrass, GC-MS, Virulence attenuation, Plant-derived antimicrobial, Biofilm, Antioxidants

## Abstract

**Background:**

The urgent need for novel antimicrobial agents arises from the escalating threat of antibiotic-resistant pathogens. Tackling the resistance mechanisms through the application of resistance modifying agents in combination to antimicrobials represents an effective strategy to combat antimicrobial resistance and to enhance the antimicrobial activity. Screening of plant extracts, essential oils and their active compounds for potential resistance modifying properties has proven effective on both a small and large scale. In this study, we define the metabolomic profile of essential oils extracted from different parts of *Cymbopogon citratus* (DC.) Stapf (lemongrass), and characterize their antioxidant and virulence attenuating activities.

**Methods:**

The major active ingredients of essential oils extracted from different parts of *Cymbopogon citratus* (lemongrass) were identified by GC-MS analysis. The minimum inhibitory concentration (MIC) of the essential oils against *P. aeruginosa* PAO1, *Staphylococcus aureus* ATCC 6538, and *Candida albicans* ATCC 10,261 was determined using the broth microdilution assay. Antibiofilm and antiprotease activities were phenotypically evaluated for the extracted lemongrass essential oils for the same standard strains and clinical isolates for the same microbes. Also, the effects on microbial virulence were validated by RT-qPCR against a subset of genes regulating biofilm, quorum sensing, and stress response in *P. aeruginosa*, and *S. aureus*. Furthermore, the antioxidant activities of the different essential oils extracted were evaluated using DPPH, β-carotene/linoleic acid and FRAP methods.

**Results:**

GC-MS analysis of the essential oils of *Cymbopogon citratus* revealed the presence of 47 different compounds distributed in the different plant organs. Active constituents such as geranial, neral, myrcene, nerolic acid, linalool, iso-citral, trans iso-citral, and neryl acetate were more abundant in the essential oil extracted from leaves (89.77%), followed by stems (82.92%) and finally roots (52.02%). *S. aureus*, and *C. albicans* were more sensitive to *C. citratus* essential oils than Gram negative *P. aeruginosa*. Incorporation of sub-MIC doses of essential oils into the culture media was sufficient to disrupt the formation of microbial biofilms in *P. aeruginosa*, *S. aureus*, and *C. albicans*, and to inactivate the proteolytic activities of microbial proteases. The essential oils of *C. citratus* showed promising and broad-spectrum biofilm eradicating activity. Sub-MIC doses of lemongrass oil dramatically reduced the expression of *relA*, *pslA*, and *spoT* in *P. aeruginosa*, and *agrA*, *icaA*, and *sigB* in *S. aureus* indicating a broad-spectrum anti-virulence activity.

**Conclusion:**

Our study identifies *C. citratus* essential oils as potential virulence-attenuating agents. Essential oils extracted from the leaves, stems, and roots of *C. citratus* exhibited significant antimicrobial, anti-biofilm, anti-virulence, and antioxidant activities, effectively combating microbial resistance. These findings suggest that *C. citratus* essential oils could be a valuable natural alternative in the fight against resistant pathogens.

**Supplementary Information:**

The online version contains supplementary material available at 10.1186/s12906-026-05431-1.

## Background

By 2050, antimicrobial resistance (AMR) could lead to the deaths of ten million people worldwide each year globally, surpassing the death rate from cancer [1–4]. Misuse and overuse of antibiotics are the main causes of AMR [5]. Moreover, microorganisms can develop a biofilm which is a community of bacterial/fungal cells enclosed in a matrix. This matrix works as a shield against the penetration of antibiotics and the cells of the host immune system [6]. Biofilm is the reason for more than 80% of microbial infections in humans [7]. The formation of biofilms by pathogens like *C. albicans* [8], *S. aureus* [9], and *P. aeruginosa* [10] poses a major challenge in medical settings. Biofilms protect these microbes from antibiotics and immune responses, making infections difficult to treat. Quorum sensing, or microbial cell-to-cell communication, plays a crucial role in regulating biofilm formation. It induces the expression of gene networks responsible for biofilm development and maintenance, allowing bacteria to coordinate their activities and establish complex, resilient communities [10–12]. Natural antimicrobials including essential oils from medicinal plants can be used not only to prevent the formation of biofilms, but also to eradicate them [13–16].

Essential oils (EOs), extracted from the different organs of plants like leaves, stems, flowers, petals or roots, are complex mixtures of volatile compounds. The yield of oil and its composition is affected by numerous factors, such as species, maturity, part of the plant, together with seasonal variations and geographical location of crops; this variability in composition hinders the analysis of EOs but on the other hand can be advantageous in case of the development of microbial resistance to EOs. Several essential oils have antimicrobial properties, mainly due to their complex chemical composition, which often includes compounds such as terpenes, phenolics, and aldehydes [17–20]. These compounds can disrupt microbial membranes, interfere with cellular processes, and inhibit microbial growth. Aside from minimal side effects, EOs exhibit broad spectrum antimicrobial activity against a wide range of pathogens, and unlike antibiotics, which target specific cellular pathways, essential oils typically have multiple mechanisms of action, making them more difficult for microbes to develop resistance. Therefore, they are either proposed as antibiotic alternatives or combined with antibiotics to have a synergistic effect against multidrug resistant microbes [5, 21, 22]. Moreover, owing to the antioxidant activity of most EOs, they can play a crucial role in combating antimicrobial resistance through various mechanisms. These include reducing oxidative stress, disrupting biofilm formation, silencing resistance mechanisms, protecting antibiotics from degradation or inactivation, and enhancing the immune response [13–20].

*Cymbopogon citratus*, popularly known as lemongrass, is an aromatic grass belonging to the family *Gramineae*. It is found in the subtropical and tropical regions of Africa, Central and South America, and Asia. *C. citratus* essential oil (CCEO) contains mainly citral, geraniol, α-oxobisabolene, and myrcene which are used as raw materials for the manufacturing of perfumes, detergents, soaps, cosmetics, beverages, and foods. Citral (a mixture of two geometrical isomers: geranial and neral) is the major constituent, which gives the oil a strong lemon fragrance. Also, it acts as a vital intermediate in the synthesis of vitamins E and A (antioxidant agents). As a result, CCEO is used in numerous pharmaceutical, cosmetic and food industries [23, 24].

Essential oil of *C. citratus* has immeasurable biological activities such as antimicrobial, antioxidant, cardio-protective, anti-carcinogenic, anti-inflammatory, anti-rheumatic, anti-diabetic, insecticidal, and anticholinesterase [16, 23, 25]. Antimicrobial activities of CCEO have been reported against *P. aeruginosa*, *Proteus vulgaris*, *Salmonella enterica*, *S. aureus*, *Mycobacterium smegmatis* and *C. albicans* [15]. Most reports on Egyptian CCEO were restricted to identify oil composition of leaves, shoots, or the whole plant. In addition, nothing has been reported on Egyptian CCEO from the roots and stems. Hence, we have performed a comparative study to characterize the metabolome of oils extracted from the leaves, roots, and stems of Egyptian *C. citratus* using GC-MS. Furthermore, the anti-virulence, antibiofilm, antimicrobial, and antioxidant activities of Egyptian CCEO extracted from the different organs were evaluated highlighting the potential of lemongrass essential oils as natural alternatives to conventional antimicrobial agents.

## Methods

### Plant material and extraction of essential oil

Fresh leaves, roots, and stems of *Cymbopogon citratus* (DC.) Stapf were collected in November 2022 from the Botanical Garden, Faculty of Pharmacy, Zagazig University, Egypt (the ease of its growth recognize its sustainability). The plant was identified by Prof. Abdel-Halim Abdel-Mogaly, Agricultural Research Centre, Ministry of Agriculture, Dokki, Giza, Egypt. Voucher specimens (Cym.cit.L for lemongrass leaves (LGL), Cym.cit.R for lemongrass roots (LGR) and Cym.cit.S for lemongrass stems (LGS)) were deposited in the herbarium of the Department of Pharmacognosy, Faculty of Pharmacy, Zagazig University, Egypt. Hydro-distillation of fresh leaves (933 g), roots (1.564 kg), and stems (1.290 kg) after their slicing was performed for each organ separately using a Clevenger-type apparatus for 3 h. The yield of extracted oils was 0.69, 0.15 and 0.28% from leaves, roots and stems of *C. citratus*, respectively. The pale-yellow oils obtained, with a strong lemon scent, were collected in dark vials, dried over anhydrous sodium sulphate, and stored in the freezer until chemical and biological evaluation.

### GC-MS analysis

A Shimadzu GC/MS-QP2020 (Kyoto, Japan) coupled to a Rtx^®^-5MS fused bonded column (30 m length, 0.25 mm internal diameter and 0.25 μm film thickness, Restek, Bellefonte, PA, USA) was utilized. The column was initially heated to 45 °C and held isothermally for 2 min, then the temperature was raised to 300 °C at a rate of 5 °C/min, and held at 300 °C for 5 min. The injector temperature was 250 °C and helium was used as a carrier gas at a flow rate of 1.41 mL/min. Mass spectra were aquired utilizing a filament emission current of 60 mA and an ionization voltage of around 70 eV. The ion source temperature was set at 200 °C, while the interface temperature was 280 °C. The oil was diluted to 1% *v/v* in hexane and 1 µl was injected at a split ratio of 1:15. Retention indices (RI) of the isolated components were calculated with respect to a set of standard n-alkanes that were analyzed separately under the same chromatographic conditions [26–28]. Identification was performed by comparing the retention index and fragmentation pattern of the resulting peaks with available literature and the NIST library 2017 [29].

### Bacterial strains, media, and chemicals

The standard strains used, *P. aeruginosa* PAO1, *S. aureus* ATCC 6538, *C. albicans* ATCC 10,261, were purchased from the Microbiological Resources Center (Cairo MIRCEN), and clinical isolates for the same microorganisms were obtained from the Department of Microbiology, Faculty of Pharmacy, Zagazig University. The antibiotic sensitivity profiles of the used standard and clinical strains have been documented in previous studies [30–33]. Microbiological media, Mueller Hinton broth (MHB), tryptone soya broth (TSB) and agar were purchased from Oxoid (Hampshire, UK). All chemicals were of pharmaceutical grade. For each experiment, the tested oil was diluted in dimethylsulfoxide (DMSO) and solubilized in the culture media using 2% *v/v* Tween 80 .

### Minimum Inhibitory Concentration (MIC)

The broth dilution method was used to determine the MIC of the reference essential oil, 2-fold serial dilutions of each oil starting from 40% *v/v* were prepared as following; a 100 µL solution containing EO diluted in DMSO (5% *v/v*) and solubilized in TSB using Tween 80 (2% *v/v*) was mixed with 100 µL TSB in a flat bottomed 96 well plate. A volume of 100 µL of TSB and 10 µL of 0.5 McFarland microbial suspension (equivalent to 5 × 10^6^ CFU/mL for bacteria and 1.4 × 10^6^ CFU/mL for fungi) in accordance with CLSI guidelines (M07-A10 for bacteria and M27-A3 for yeasts) was then added to each well and the plate was incubated at 37 °C for 24 h. Positive growth control wells contained DMSO (5%), Tween 80 (2%) and microbial suspension in TSB, and control wells contained fungal suspensions and fluconazole (25 µg/µL) or bacterial suspension and meropenam (2 µg/µL). The MIC was calculated as the lowest concentration of each fraction that inhibited the visible growth (turbidity) according to the literature [34–36].

### Antibiofilm activity

Biofilm formation was quantified as previously described [26, 37]. Briefly, bacterial and yeast overnight cultures were prepared in TSB and diluted with TSB to an optical density OD600 nm of 0.4. Aliquots (10 µL) of the optically adjusted bacterial suspensions were added each to 10 mL of fresh TSB containing the appropriate fraction (in 1/8th MIC doses). The plates were incubated at 37 °C for 24 h. The planktonic cells were aspirated, and the plates were washed 3 times with 2 mL sterile water and then allowed to dry. The adherent bacterial cells were fixed with absolute methanol for 25 min and then stained with crystal violet (1%) for a further 20 min at room temperature. Excess stain was washed with water, adherent stain was dissolved in acetic acid (33%), and absorbance measured at 590 nm using a Biotek Spectrofluorometer (Biotek, Winooski, VT, USA) according to Cruz et al. [38]. The assay was performed in triplicate and absorbance was shown as the mean ± standard error the percentage change from untreated controls (Microorganisms grown on medium only).

### Biofilm eradication assay

Microbial strains were grown under ideal conditions to form biofilm as previously reported [26], tubes containing the preformed intact biofilm of each microbe were treated with 1% *v/v* of each oil (diluted in DMSO (5% *v/v*) and solubilized with Tween 80 (2% *v/v*)) or with DMSO (5% *v/v*) in Tween 80 (2% *v/v*) (as a negative control). The biofilms were then incubated for 1 h at 37 °C, fixed and stained as previously described, dissolved in acetic acid (33%) according to the literature [39] and the absorbance was measured at 590 nm according to Chen et al. [40]. The biofilm eradication percentage (%) was calculated:

$$\begin{aligned} \mathrm{Biofilm}\;\mathrm{eradication}\;\%=& [(\mathrm{Absorbance}\;\mathrm{of}\;\mathrm{control}\;\mathrm{at}\;\mathrm{OD}590\;\mathrm{nm}\\&-\mathrm{Absorbance}\;\mathrm{of}\;\mathrm{test}\;\mathrm{at}\;\mathrm{OD}590\;\mathrm{nm}) \\& /\mathrm{Absorbance}\;\mathrm{of}\;\mathrm{control}\;\mathrm{at}\;\mathrm{OD}590\;\mathrm{nm})]\times100 \end{aligned}$$

### Antiprotease activity

In order to evaluate the inhibitory effect of each fraction on protease activity, the skim milk agar method was employed as previously described [26]. Overnight cultures in MH broth with the referred fraction (1/8th MIC dose) were centrifuged at 9500× g for 20 min at room temperature. Aliquots (100 µL) of the supernatants were added to the wells of 5% skim milk agar plates, incubated overnight at 37 °C, and the clear zones formed around the wells were measured. The test was performed in triplicate and the obtained results were presented as the mean ± standard error of the mean for the zone of inhibition.

### Quantitative reverse transcriptase polymerase chain reaction RT-PCR

RT-PCR was employed to evaluate the expression of biofilm-related genes (*relA*, *rsmZ*, *pslA*, and *spoT*) in *P. aeruginosa* PAO1, and (*agrA*, *lcaA*, and *sigB*) in *S. aureus* both before and after 24 h exposure to LGL oil tested at sub-MIC doses (1/8th MIC). RNA extraction from samples was carried out using the QIAamp Rneasy Mini kit (Qiagen, Hilden, Germany, GmbH). For each sample, 200 µL was combined with 600 µL of RLT buffer, containing 10 µL β-mercaptoethanol per 1 mL, and incubated at room temperature for 10 min. Subsequently, one volume of 70% ethanol was added to the lysate, and the procedure was completed following the Total RNA Purification protocol of the QIAamp Rneasy Mini Kit (Qiagen, Hilden, Germany, GmbH).

The primers used were supplied by Metabion (Germany) (Table 1). In a 25 µL reaction, primers were included, with each containing 10 µL of the 2× HERA SYBR^®^ Green RT-qPCR Master Mix (Willowfort, UK), 1 µL of RT Enzyme Mix (20x), 0.5 µL of each primer at a 20 pmol concentration, 5 µL of water, and 3 µL of RNA template [5]. The reaction was conducted using a StepOne real-time PCR machine, and amplification curves and Ct values were determined by the StepOne software [5].

**Table 1:** Primers used for qRT‑PCR analysis

Gene name	Primer sequence (5’-3’)	Ref.
*agrA* (F)	TGATAATCC TTATGAGGTGCTT	[37]
*agrA* (R)	CAC TGTGAC TCG TAACGA AAA	[37]
*icaA* (F)	CTGGCGCAGTCAATACTATTTCGGGTG TCT	[37]
*icaA* (R)	GACCTCCCAATGTTTCTGGAACCA ACATCC	[37]
*sigB* (F)	CGTCTCGGA ACATGTACACTCCAAG	[37]
*sigB* (R)	GTCCTT TGA ACG GAAGTT TGA AGCC	[37]
*relA* (F)	CGC GAT CCG TGC CTA TG	[34]
*relA* (R)	CAG AAC GTT GAT CCG CTC ATT	[34]
*spoT* (F)	AAG GCG TTC AAC GAG ATC ATG	[34]
*spoT* (R)	CCC AGT ACG CGAT AGC AGG TA	[34]
*pslA* (F)	TCCCTACCTCAGCAGCAAGC	[34]
*pslA* (R)	TGTTGTAGCCGTAGCGTTTCTG	[34]
*16 S rRNA* (F)	AGAGTTTGATCCTGGCTCAG	[34]
*16 S rRNA* (R)	ctacggctaccttgttacga	[34]

*F* Forward, *R* Reverse

To assess the variation in gene expression among the RNA samples, the Ct of each sample was compared to that of the positive control group using the “ΔΔCt” method outlined by [5], yielding the following ratio: (2^−ΔΔct^).

### Antioxidant activity

#### DPPH radical scavenging activity assay

The 2,2-Diphenyl-1-picrylhydrazyl (DPPH) radical scavenging activity was determined by the method of Ramadan et al., and Göçer and Gülçin [41, 42]. The oils were dissolved in DMSO and one mL of each sample at different concentrations (100–3200 µg/mL) was combined with four mL of 0.15 mM DPPH (in 95% ethanol) and vortexed vigorously. The reaction mixtures were incubated in the dark at room temperature for 30 min before measuring the absorbance at 517 nm. A control (a reaction containing DPPH solution and DMSO without oil sample) was also recorded. The radical scavenging capacity of the samples was estimated as the decrease in absorbance of the DPPH radicals and calculated using the equation:

$$\begin{aligned}\mathrm{Rad}&\mathrm{ical}\;\mathrm{scavenging}\;\mathrm{activity}\;\left(\%\right)\\&=\left[\left(\mathrm A\;\mathrm{control}-\mathrm A\;\mathrm{sample}\right)/\mathrm A\;\mathrm{control}\right]\times100\end{aligned}$$

$$\mathrm A=\mathrm{absorbance}\;\mathrm{at}\;517\;\mathrm{nm}$$

#### β-carotene /linoleic acid bleaching method

The capacity of *C. citratus* essential oils to inhibit the bleaching of β-carotene was examined as reported by Dastmalchi et al. [21]. An amount (0.2 mg) of β -carotene in 1 mL chloroform, 20 mg of linoleic acid, and 200 mg of Tween 20 were well mixed in a round-bottom flask. After removing the chloroform, 50 mL of distilled water were added, and the mixture was stirred vigorously. Three mL of emulsion aliquots were distributed in tubes containing the tested samples at concentrations of 100–3200 µg/mL. After mixing, 0.5 mL of each sample was pipetted into a cuvette, and the absorbance was recorded at 470 nm. The remaining samples were placed in a 50 °C water bath for 120 min and the absorbance was recorded at 470 nm. Finally, a control without an added sample was assessed. The protection index (PI) was calculated according to the following equation:

$$\mathrm{Protection}\;\mathrm{index}\;\left(\%\right)=\mathrm A/\mathrm A0\times100$$

Where: A0 sample is the absorbance of the sample at time - zero, A is the absorbance after 120 min. 

#### Ferric Reducing Antioxidant Power (FRAP) assay

The reducing power of *C. citratus* essential oils were estimated by measuring the absorption of Perl’s Prussian blue complex resulting from reducing Fe^+ 3^ to Fe^+ 2^ at 700 nm, according to the previously described method [41, 43]. Ten parts of 0.3 M acetate buffer (pH 3.6), one part of 10 mM 2,4,6-tripyridyl-s-triazine (TPTZ) in 40 mM HCl, and one part of 20 mM FeCl_3_.6H_2_O in distilled water were combined to prepare the FRAP reagent. Three mL of FRAP reagent and 0.1 mL of the DMSO essential oil solution were combined to start the reaction. The reaction was carried out for 10 min at 37 °C in the dark and the absorbance at 593 nm was measured in comparison with a blank prepared with DMSO.

### Statistical analysis

All experimental data were expressed as mean ± standard error of the mean (SEM) from at least three independent replicates. Comparisons between treated and control groups were conducted using One way analysis of variance (ANOVA) followed by the suitable post hoc test test unless/otherwise mentioned based on the study design. A *p*-value of < 0.05 was considered statistically significant. Statistical analyses and graphical outputs were performed using GraphPad Prism version [5.0] (GraphPad Software Inc., San Diego, CA, USA).

## Results

### GC-MS analyses of essential oils extracted from different organs of *C. citratus*

GC-MS analyses of essential oil from different organs of *C. citratus* resulted in the identification of 19 compounds from the leaves, 37 compounds from the roots and 16 compounds from the stems, representing 96.85%, 90.71% and 95.58% of the total oils contents in the investigated organs, respectively (Table 2 & Additional file 1: Figure S1).

**Table 2:** Chemical composition of *Cymbopogon citratus*’s essential oils based on GC-MS analyses

No	Compound	Leaves			Roots			Stems		
		Rt	RI	Area %	Rt	RI	Area %	Rt	RI	Area %
1	6-Methyl hept-5-en-2one	8.645	959	1.02				8.645	959	0.68
2	Myrcene	9.265	979	8.03	9.25	979	0.71	9.24	979	2.96
3	B-cis-Ocimene	10.61	1023	0.24						
4	2-Methyl-6-methylene-2-Octene	10.735	1027	0.19						
5	Linalool	12.375	1079	1.3	12.385	1080	0.32	12.37	1079	0.92
6	7-methyl-3-methylene-6-octenal	13.535	1117	0.81				13.53	1117	0.46
7	Citronellal	13.81	1126	0.21						
8	Isocitral	14.135	1136	1.51	14.14	1136	0.3	14.13	1136	0.99
9	Borneol				14.26	1140	0.08			
10	trans-Isocitral	14.665	1154	2.28	14.67	1154	0.46	14.655	1153	1.54
11	Neral	16.445	1212	33.13	16.52	1215	19.19	16.4	1211	34.2
12	Piperitone	16.635	1219	0.25	16.69	1220	0.33	16.605	1218	0.56
13	Geraniol							17.055	1233	5.5
14	Geranial	17.325	1242	41.23	17.345	1243	30.87	17.28	1241	41.54
15	4,8-Dimethyl-3,7-nonadien-2-one	17.485	1248	0.27				17.465	1247	0.61
16	2-Undecanone	18.075	1268	0.51						
17	z- Octenol propanoate	18.795	1293	1.23	18.825	1294	1	18.78	1292	1.34
18	8-hydroxy-neo-menthol	19.75	1327	1.89	19.78	1328	1.32	19.74	1326	1.82
19	Nerolic acid	20.005	1336	1.87				20.015	1336	0.77
20	Ethylnerolate				20.295	1346	2.33			
21	Neryl acetate	20.51	1354	0.42	20.52	1355	0.17			
22	b- elemene				21.17	1378	0.23			
23	E-caryophyllene				21.895	1405	0.41			
24	α-trans-bergamotene				22.375	1423	0.66			
25	α -Humulene				22.75	1437	0.36			
26	α -Himachalene				23.315	1459	0.14			
27	7-epi- α -Selinene				23.49	1466	0.27			
28	2-Tridecanone	23.565	1469	0.46						
29	b-Selinene				23.575	1469	0.24			
30	epi-Cubebol				23.75	1476	0.86			
31	Cubebol				24.25	1495	1.54			
32	δ-Cadinene				24.465	1504	0.57			
33	10-epi-Cubebol				24.59	1510	0.19			
34	Hedycaryol				24.94	1528	0.34			
35	Caryophyllene oxide				25.75	1569	0.43			
36	5-epi-7-epi- α -eudesmol				26.365	1600	1.28			
37	α -Selin-6-en-4-ol				26.705	1612	9.97	26.565	1607	1.05
38	1-epi-Cubenol				26.905	1619	0.29			
39	Eremoligenol				27.035	1624	0.96			
40	epi- α -Muurolol				27.205	1630	3.5	27.405	1637	0.64
41	α -Cadinol				27.505	1640	6.82			
42	Citronellyl ester				28.1	1661	0.65			
43	Eudesm-7(11)-en-4-ol				28.365	1671	1.46			
44	2E,6Z-Farnesal				28.675	1682	0.21			
45	2Z,6E-Farnesol isomer)				28.97	1692	1.3			
46	2E,6E-Farnesal				29.27	1703	0.49			
47	2- α -acetoxy-Amorpha-4,7(11)-diene				30.9	1776	0.46			
	Total percentage			96.85			90.71			95.58

Retention time (RT) in minutes, and Retention indices (RI)

**Fig. 1:**
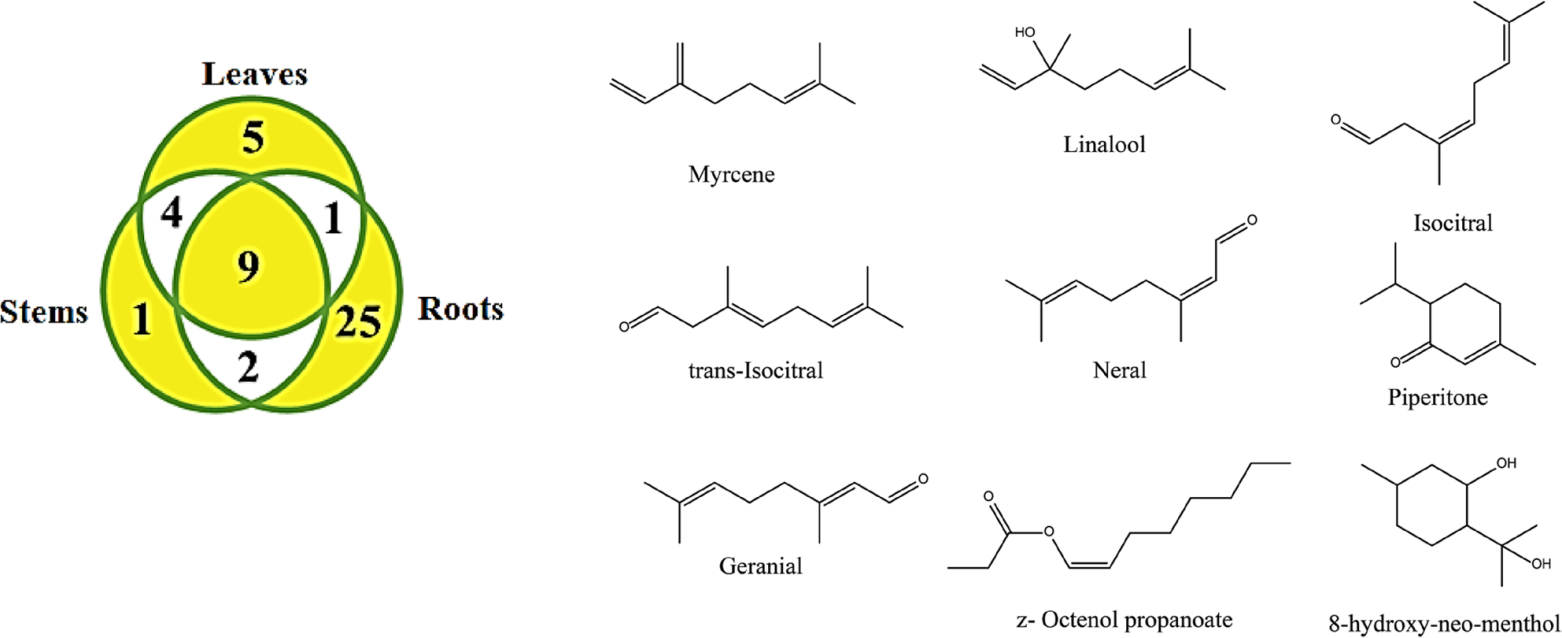
Van diagram of *Cymbopogon citratus*’s essential oil components and chemical structure of shared 9 compounds

It was obvious from the results that geranial is the major oil component in the three organs with percentages of 41.23%, 30.87% and 41.54% in the leaves, roots and stems, respectively followed by neral with percentages of 33.13%, 19.19% and 34.2% in the same oils. The numbers of sharing oil contents between the three parts are shown in Fig. 1. Other main components that represent more than 2% of the oil’s composition in each organ include myrcene and trans-isocitral in the leaves, α-selin-6-en-4-ol, α-cadinol, epi- α-muurolol and ethylnerolate in the roots and geraniol and myrcene in the stems.

### MIC and antibiofilm activity

**Fig. 2:**
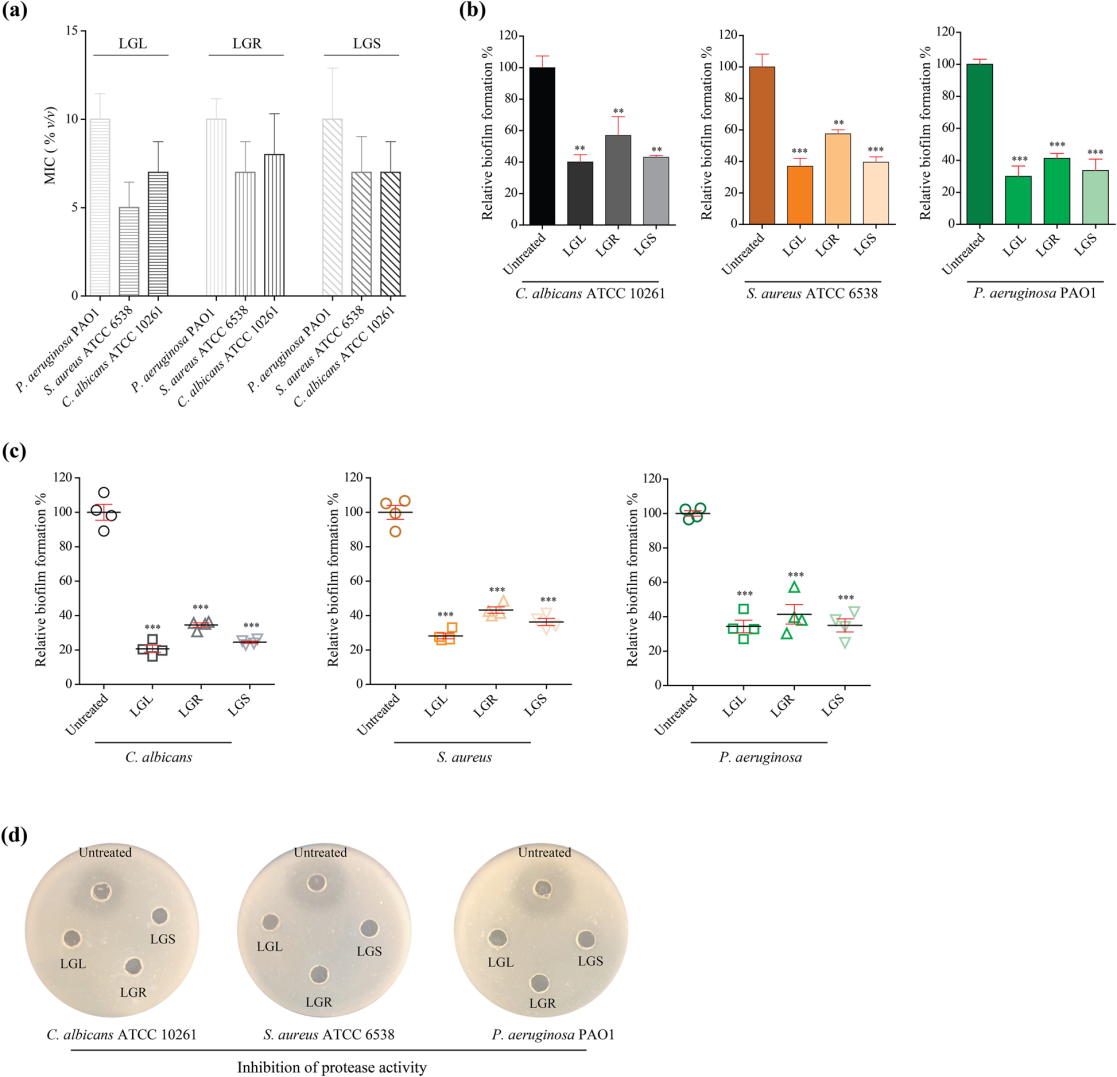
Antimicrobial activity of *C. citratus* essential oils extracted from leaves (LGL), roots (LGR), and stems (LGS) against *P. aeruginosa*, *S. aureus*, and *C. albicans*. **a** Minimum inhibitory concentration (MIC) **b** Antibiofilm activity against standard strains *P. aeruginosa* PAO1, *S. aureus* ATCC 6538, and *C. albicans* ATCC 10,261 exposed to sub-MIC doses of each oil relative to untreated cultures. **c** Antibiofilm activity against clinical isolates of *P. aeruginosa*,* S. aureus*, and *C. albicans*. **d** Antiprotease activity. Data are presented as mean ± standard error of the mean (SEM) of three independent replicates in case of standard strains or four independent replicates in case of clinical isolates. In 2b, and 2c, data were statistically analyzed compared to untreated using One ANOVA followed by Dunnett’s test, ** *p* < 0.01, and *** *p* < 0.001

For the microbiological evaluation of lemongrass essential oils, we started with defining the minimum inhibitory concentration (MIC) of each oil against three selected microbes *P. aeruginosa* PAO1, *S. aureus* ATCC 6538, and *C. albicans* ATCC 10,261. LGL has MICs of 10 ± 1.44, 5 ± 1.44, and 7 ± 1.73% (*v/v*) against *P. aeruginosa*, *S. aureus*, and *C. albicans* respectively. LGR showed MICs of 10 ± 1.15, 7 ± 1.73, and 8 ± 2.31% (*v/v*), while LGS showed MICs of 10 ± 2.89, 7 ± 2.02, and 7 ± 1.73% (*v/v*) against the same microbes (Fig. 2a).

We then examined the ability of lemongrass essential oils to disrupt the formation of microbial biofilms. The microbial cultures were grown in the presence of sub-MIC doses (1/8th of the MIC) of each oil for each microbe, the biofilm of *P. aeruginosa* lost 70, 59, and 66.3% of its density when grown in the presence of sub-MIC dose (1.2% v/v) of LGL, LGR, and LGS oils respectively, the biofilm density of *S. aureus* decreased by 63, 42.5, and 60.5% upon exposure to sub MIC doses of LGL (0.625% v/v), LGR (0.875% v/v), and LGS (0.875% v/v) oils respectively, while the ability of *C. albicans* to form an intact biofilm decreased by 60, 43, and 57% upon exposure to sub-MIC dose (0.875% v/v) of LGL, LGR, and LGS oils respectively (Fig. 2b). In the same context, we evaluated the biofilm inhibitory actions of the 3 essential oils against clinical isolates of *P. aeruginosa*, *S. aureus*, and *C. albicans*, superior antibiofilm activity was observed for LGL, followed by LGS, and LGR respectively (Fig. 2c).

The lemongrass oils not only hindered the ability of microbial cells to construct biofilm but also reduced pre-formed structure, the three essential oils were able to dissolve the formed biofilms of the different microbes. At a 1% *v/v* concentration, LGL oil removed 78, 72, and 85% of the formed biofilms from *P. aeruginosa*, *S. aureus*, and *C. albicans* respectively, LGR oil removed 74, 69, and 77% of the formed biofilms, while LGS oil removed 73, 67, and 79% of the formed biofilms from the same microorganisms.

### Antiprotease activity

The ability of lemongrass essential oils to inactivate microbial proteases was checked. Microbial lysates from cultures treated with sub lethal doses of each oil for each microbe were tested for protease activity using skimmed agar plates. All the lysates from treated cultures showed no significant inhibition zone around the wells indicating inactivation of microbial proteases compared to control lysates where clear inhibition zones 2.7 cm ± 0.1, 3.4 cm ± 0.07, and 4 cm ± 0.16 in diameter were detected for *C. albicans*, *S. aureus*, and *P. aeruginosa* respectively, confirming lysis of milk casein with microbial proteases (Fig. 2d & See uncropped images in Additional file 2: Figure S2 ).

### Gene expression analysis of virulence-associated genes by RT-qPCR

Next, we analyzed the antivirulence properties of lemongrass leaves oil (LGL) by measuring the expression of genes associated with the regulatory networks of transition from planktonic to biofilm growth, stress adaptation, and quorum-sensing in *P. aeruginosa* and *S. aureus* before and after treatment with LGL essential oil.

**Fig. 3:**
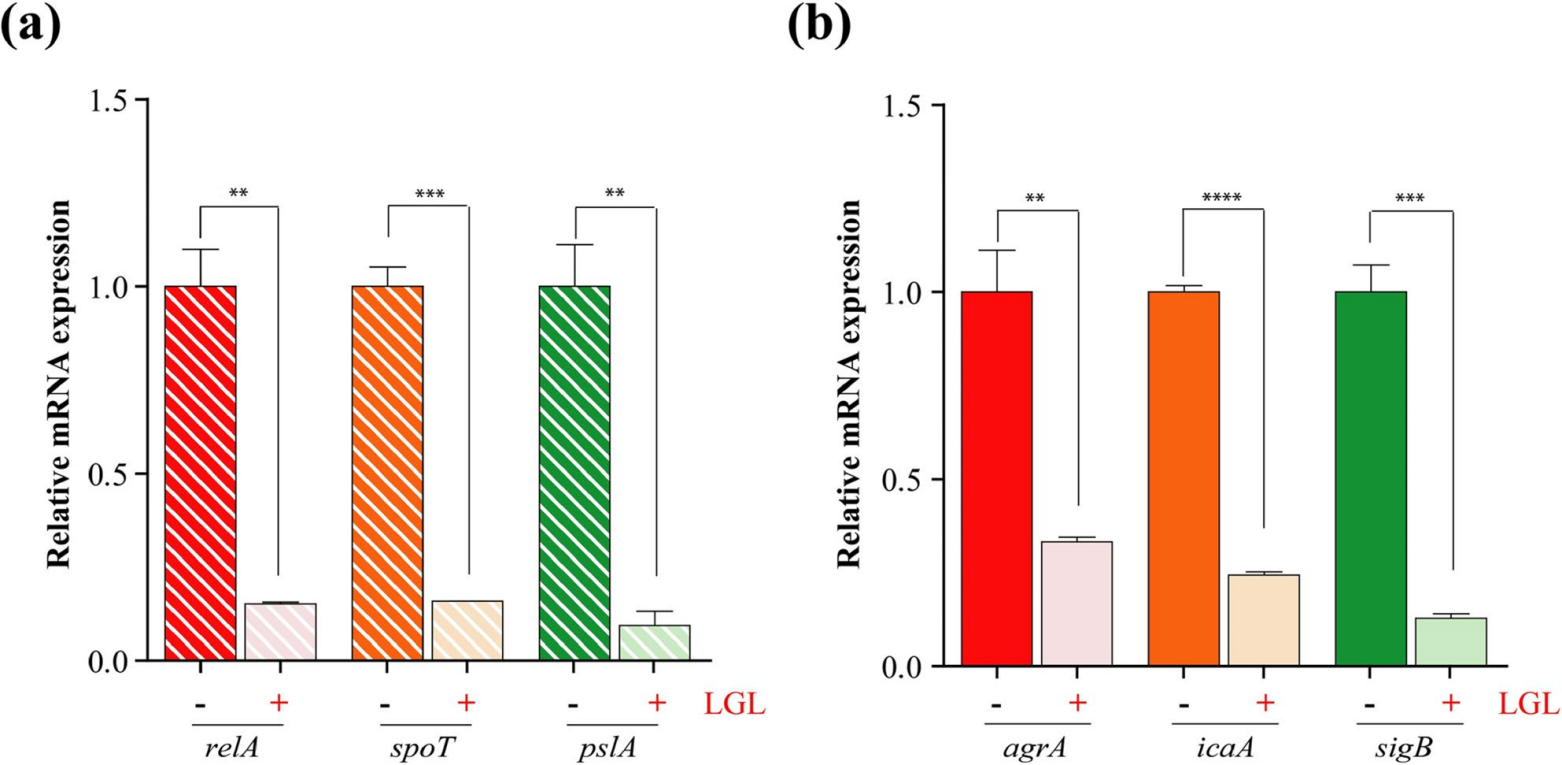
Antivirulence activity of LGL essential oil against *P. aeruginosa*, and *S. aureus* assessed by RT-qPCR. (**a**) qPCR analysis of *P. aeruginosa* virulence genes in presence (+) or absence (-) of LGL essential oil (**b**) qPCR analysis of *S. aureus* virulence genes in presence or absence of LGL essential oil. The data represent the mRNA expression of each gene relative to *16 S rRNA* (housekeeping) then normalized to the untreated control of the same gene. Results are expressed as Mean ± SEM from three independent experiments. (-) without oil, (+) with oil, data were statistically analyzed using unpaired Student’s T test, ** *p* < 0.01, *** *p* < 0.001, and **** *p* < 0.0001

RT-qPCR demonstrated that upon exposing *P. aeruginosa* to sub-MIC dose of LGL (1/8th MIC), there was a notable and significant reduction in the expression of *relA*, *pslA*, and *spoT*. The effect was particularly prominent on *pslA*, which plays a crucial role in biofilm formation [44] as depicted in Fig. 3a. On the other hand, lemongrass oil significantly altered the expression of *agrA*, *icaA*, and *sigB*, with a particularly pronounced effect on *sigB* which mediates biofilm formation [45] in *S. aureus*, as illustrated in Fig. 3b.

Collectively, our findings establish a connection between the broad-spectrum antivirulence activity of lemongrass oil and its exceptional capacity to alter the expression of the regulatory genes.

### Antioxidant activity

**Fig. 4:**
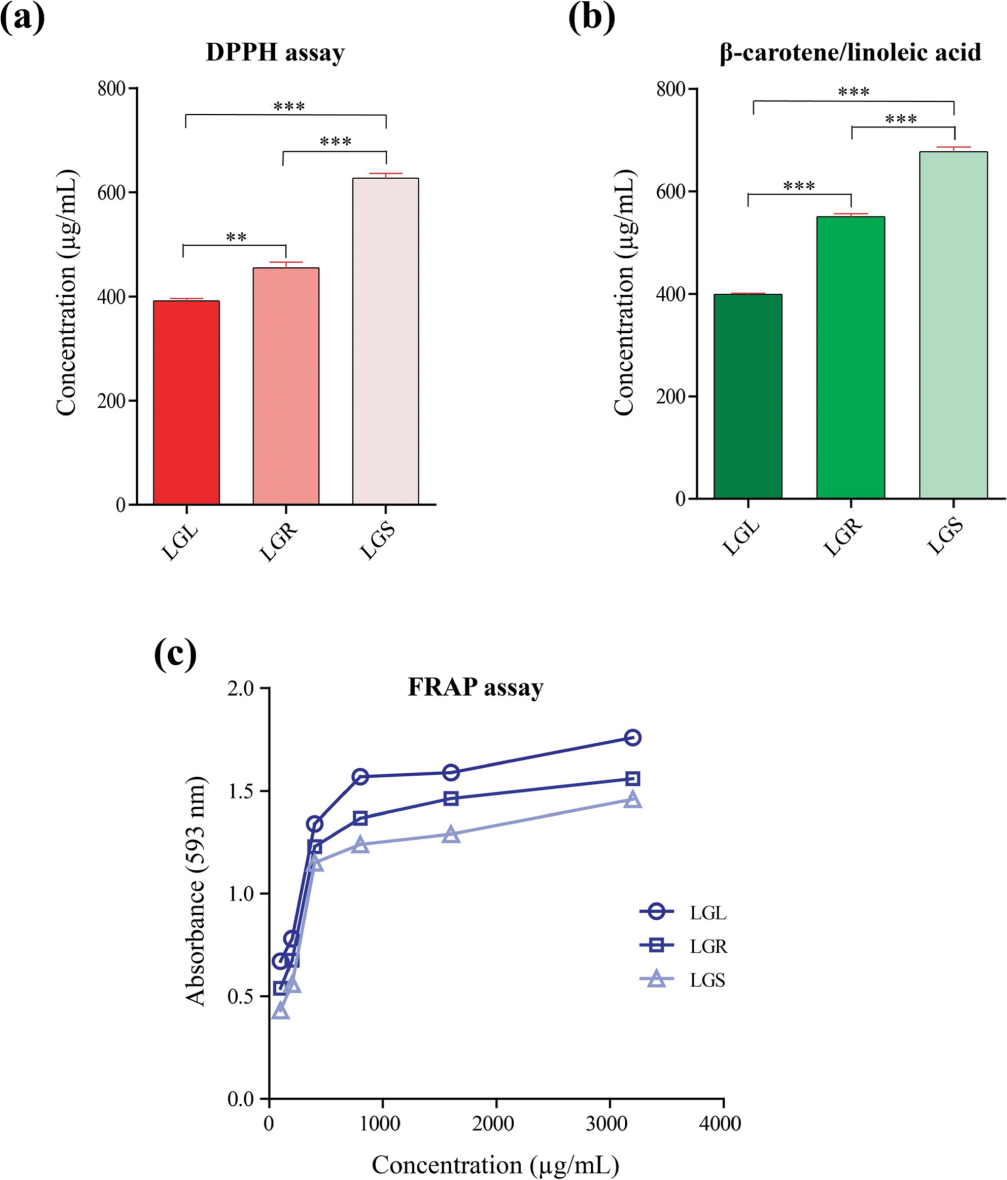
Antioxidant activity of *C. citratus* essential oils from leaves (LGL), roots (LGR) and stems (LGS) respectively. **a** IC_50_ of each oil measured by DPPH assay. **b** PI_50_ of each oil measured by β-carotene/linoleic acid method. **c** FRAP assay. Data represented as average of 3 independent experiments ± SEM. Data were statistically analyzed using One way ANOVA followed by Tukey’s Test, ** *p* < 0.01, and *** *p* < 0.001

Antioxidant activities of the different *C. citratus* essential oils were evaluated using different DPPH, β-carotene/linoleic acid and FRAP methods. When we analyzed the antioxidant activity of different oils using DPPH reagent and measured the IC_50_ (the oil concentration that scavenge 50% of the DPPH radicals), the results indicated that LGL is the most active oil as a free radical scavenging agent with IC_50_ of 391.5 ± 4.5 µg/mL followed by LGR and LGS with IC_50_ 454.9 ± 10.75 µg/mL and 627.24 ± 9.21 µg/mL, respectively (Fig. 4a). Similar results were indicated from the oils’ ability to protect β-carotene from bleaching with protection index (PI_50_) of 399.1 ± 1.9 µg/mL, 550.57 ± 5.94 µg/mL and 677.44 ± 9.06 µg/mL for oils of leaves, roots and stems, respectively (Fig. 4b). The oils were able to reduce ferric ions in a dose dependent manner with best activities for LGL followed by LGR and then LGS upon comparing the same dose (Fig. 4c).

## Discussion

The formation of microbial biofilms poses a significant health problem, as they play a crucial role in microbial resistance to antibiotics by acting as physical barriers. Biofilms exhibit elevated tolerance to antibiotics compared to planktonic cells due to hindered antibiotic penetration, differentiated microbial metabolism, and varied growth states within the biofilm structure. Consequently, antibiotics often fail to affect certain populations of cells within biofilms, which also facilitate a higher rate of resistance gene transfer [46]. These biofilms are formed in response to microbial quorum sensing system activation [47]. One of the main methods to overcome this phenomenon is targeting microbial resistance mechanism *via* impairing their quorum sensing system and consequently microbial virulence factors. This could affect microbial cell communication system and perturb their biofilm formation as well as their motility, protease, and hemolytic ability [48–51].

The high toxicity of many chemically synthesized anti-biofilm or anti-quorum sensing agents during clinical stages significantly limits the development and use of these antimicrobials [52]. However, natural products that are effective as antibiofilm and anti-quorum sensing agents and, simultaneously, safe are promising alternatives [53, 54]. Several essential oils have been proven to be effective as anti-biofilm and quorum sensing inhibitors including essential oils of caraway, cassia, balsam Peru, marjoram, rosemary, thyme, lavender, sage, pignut, savory, coriander, cumin, bergamot, and red thyme [55–60].

Oils from the same plant species could show changes in their chemical composition due to several factors such as growth condition, collection time and country of origin. Moreover, the composition of the oil from the different parts of the same plant may be different because of the theory of biosynthesis, transportation and storage of secondary metabolites [61, 62]. This variation could affect reproducibility and should be minimized specially on the clinical levels, that is why its important to adhere to plant collection place and time and perform a standardization study for any complex natural product before clinical use. In the present study, we defined the chemical components of lemongrass essential oil from leaves, roots and stems of the Egyptian plant by GC-MS analysis. Nineteen, thirty-seven and sixteen compounds were identified from leaves, roots and stems, respectively. The identified compounds cover broad spectrum of hydrocarbons, oxygenated and non-oxygenated terpenes with geranial and neral as major components. This result is in agreement with the reported major components of leaves [16, 63, 64], shoots [64, 65], and the whole plant [66] of the Egyptian CCEO. Similarly, geranial and neral are the majors of EO components of other species of *Cymbopogon* [67]. Contrary to the previous, main components of EOs of *C. citratus*’s roots from Nepal were α-elemol and geranial. Additionally, α-elemol and geraniol were the predominant EO components of *C. winterianus*’s roots and leaves, respectively [68]. Furthermore, EOs of *C. flexuosus*, *C. pendulus* and *C. khasianus* were reported to be rich in elemol [69].

Previous reports revealed the anti-biofilm activity of *Cymbopogon flexuosus* essential oil against *P. aeruginosa*, *Salmonella typhimurium* [70], and *S. aureus* [71]. Similarly, *C. citratus* was found to inhibit biofilm of different microorganisms such as *Streptococcus*, *Staphylococcus*, *Lactobacillus*,* P. aeruginosa*, and *Candida* species [72–76]. *P. aeruginosa*, *S. aureus* and *C. albicans* are representative examples for Gram-negative bacteria, Gram positive bacteria and fungi, respectively that cause outbreak infections in hospitals due to their arms of virulence including their ability to form biofilms and consequently, development of resistance against antibiotics [77, 78]. No previous report dealt with evaluating *C. citratus* essential oils from leaves, roots and stems against *P. aeruginosa*, *S. aureus* and *C. albicans* except Narkar et al. [76], that reported the antibiofilm activity of *C. citratus* essential oil from leaves and stems against *P. aeruginosa*. In accordance to Narkar et al. [76], gram positive bacteria (*S. aureus*) and fungi (*C. albicans*) were more sensitive (lower MIC) to *C. citratus* essential oils than gram negative *P. aeruginosa*.

**Fig. 5:**
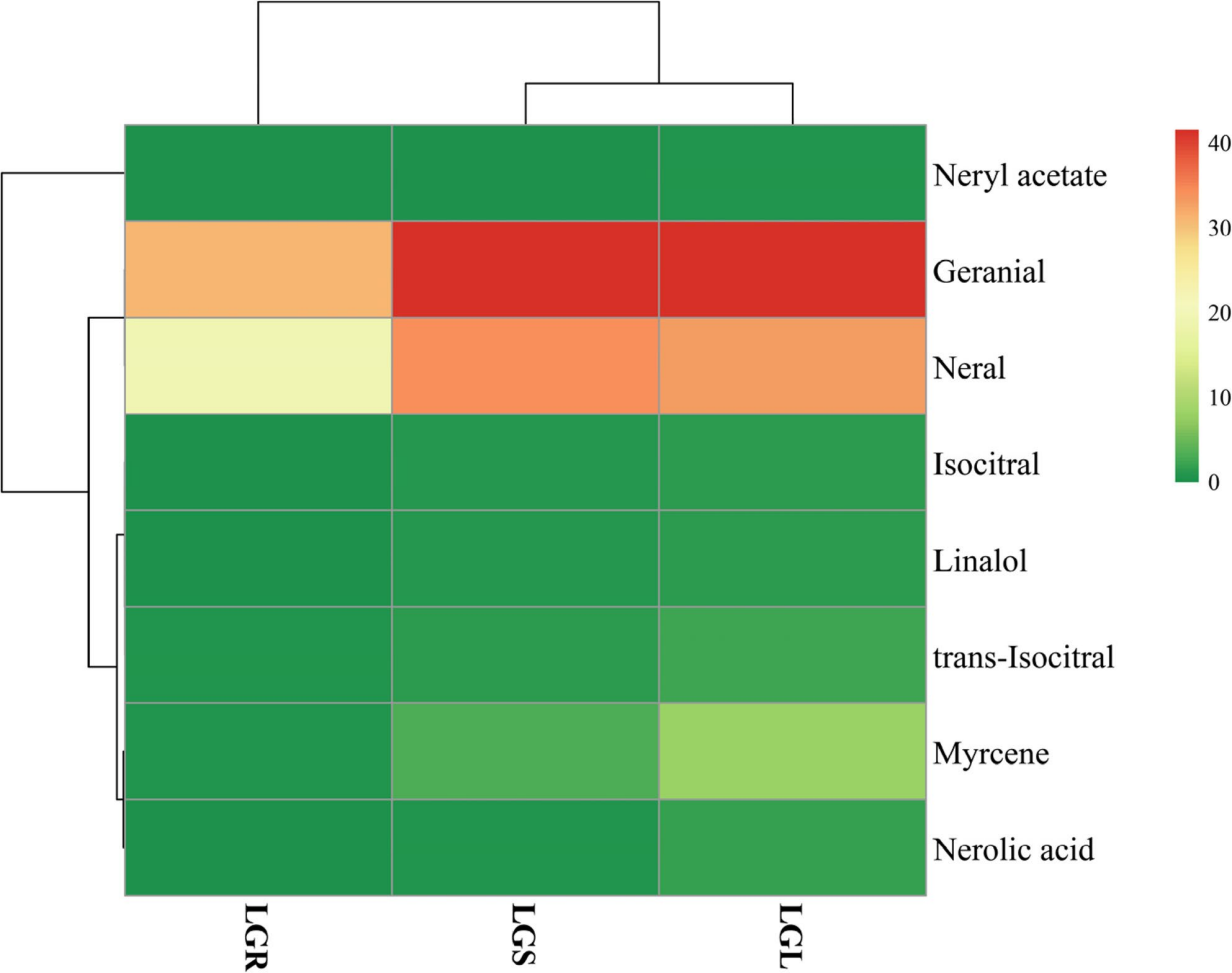
Heatmap depicting the abundance of selected active constituents with reported antimicrobial activities within the essential oils extracted from leaves (LGL), stems (LGS), and roots (LGR) of *C. citratus*. Heatmap was created using ClustVis online tool [83], based on data retrieved from Table 2

No reports dealt with identifying essential oil components of Egyptian *C. citratus*’s roots and stems. In the current study, the essential oils from the different parts of the plant under investigation at a dose of 1/8th MIC were able to decrease density of microbial biofilms of tested microorganisms in vitro in the following order; leaves more than stems more than roots. A possible explanation for the differential activity among the various *C. citratus*’s essential oils could be attributed to the variable concentrations of active constituents known for their antimicrobial properties such as geranial, neral, myrcene, nerolic acid, linalool, isocitral, trans-isocitral, and neryl acetate [15, 79–81], the sum of concentrations of these active compounds surprisingly follows the order of observed antivirulence activity, being most abundant in the leaves, followed by the stems, and finally the roots (Fig. 5). For instance, the concentration of geranial in LGS and LGL essential oils is approximately 1.37 times higher than in LGR. Similarly, neral concentrations in LGS and LGL essential oils are about 1.79 times higher than that in LGR. This higher concentration of these antimicrobial compounds in the leaves and stems compared to the roots may account for the enhanced antimicrobial efficacy observed in essential oils extracted from these parts of the plant. Another possibility could be specific synergism existing among the distinct cocktails of active constituents in each oil from each part which may create extraordinary antibiofilm action mediated by myrcene for example [80], or linalool [82]. Taken together, this gradient in active constituent levels likely explains the superior antimicrobial efficacy of lemongrass essential oils derived from the leaves compared to those from the stems and roots.

Interestingly, the oils at the same concentration were able to inhibit microbial proteases in the tested microorganisms indicating their ability to inhibit a common virulence factor in bacteria and fungi. Microbial proteases can cause necrosis, tissue damage and digestion of plasma proteins in infected hosts [84].

Going from phenotypic analysis, our study delved into the molecular underpinnings of the remarkable anti-virulence activity exhibited by lemongrass essential oil. Exposure to sub-inhibitory doses of LGL essential oil induced notable alterations in the expression of genes involved in biofilm formation (*icaA*, and *agrA*) in case of *S. aureus* [37], and (*relA*, and *pslA*) in case of *P. aeruginosa* [85–87], in addition to genes regulating quorum sensing and response to stress (*sigB*) in case of *S. aureus* [37], and (*spoT*) in case of *P. aeruginosa* [85]. Together, our results highlight a link between the broad-spectrum anti-virulence properties of lemongrass oil and its ability to disrupt the gene circuits that govern microbial biofilm.

Aside from their potential anti-virulence activity, essential oils of *C. citratus* could combat microbial resistance through their antioxidant properties that were indicated by their ability to scavenger free radicals, protect β-carotene bleaching and ferric ion reduction using the DPPH, β-carotene/linoleic acid and FRAP methods, respectively.

Antioxidants have been repurposed as antibiofilm agents by targeting multiple stages of biofilm formation, including the inhibition of initial adhesion and suppression of oxidative stress-induced biofilm development [88–90]. In this aspect, LGL possessed the superior antioxidant activity followed by LGR and LGS, which matches the superior antibiofilm activities of the same oil. On the other hand, antioxidants play a key factor in overcoming microbial resistance, this could be attributed to inhibition of ATP production *via* the neutralization of Reactive Oxygen Species (ROS) resulting in microbicidal action [91]. As a result, we represent *C. citratus* essential oils as potential anti-virulence agents of natural origin against *P. aeruginosa*, *S. aureus* and *C. albicans*.

## Conclusions

The essential oils of *Cymbopogon citratus*, derived from leaves, roots, and stems, were analyzed using GC-MS, identifying a total of forty-seven compounds, with the highest concentration observed in the leaf oil. Microbiological evaluations demonstrated that these oils effectively inhibit biofilm formation of *P. aeruginosa*, *S. aureus*, and *C. albicans*, while also inhibiting the protease activity of these pathogens. Furthermore, the oils exhibited remarkable antioxidant activity. This study highlights the potential of *C. citratus* essential oils as broad-spectrum virulence-attenuating agents and suggests future in vivo studies for treating biofilm-associated infections (e.g., wound or skin infection) and catheter-associated infections. The inclusion of oils extracted from various parts of the plant (leaves, roots, and stems) provides new and exciting perspectives on the overall antimicrobial potential of this plant.

### Limitations

The antimicrobial efficacy of lemongrass (*Cymbopogon citratus*) essential oil is influenced by variability in its chemical composition, concentration-dependent activity, and sensitivity to environmental factors. Although the oil demonstrated notable antimicrobial and antibiofilm effects, higher concentrations may cause skin irritation, and standardized guidelines ensuring consistent quality and efficacy are lacking. This study focused on evaluating the overall activity of the complete essential oil mixture; however, the individual and interactive effects of its major constituents (citral, myrcene, and linalool) were not assessed. In vivo investigations are also required to confirm its clinical relevance and to explore potential synergism with conventional antibiotics, particularly in infection models such as skin infections. Future work should therefore aim to standardize formulations, isolate and test key compounds individually and in combination, and establish safety and efficacy profiles to support potential therapeutic applications.

### Future prospects

Potential advancements in nanotechnology, drug delivery systems, synergistic formulations, and dosage forms can significantly enhance the stability, efficacy, and safety of lemongrass essential oil. These developments could expand its practical application in biofilm associated infections.

## Supplementary Information

Supplementary Material 1.

Supplementary Material 2.

## Data Availability

The datasets used and/or analysed during the current study available from the corresponding author on reasonable request.
