# Supplementary material for: Chemical profile, anti-biofilm and antioxidant activities of Cymbopogon citratus (DC.) Stapf essential oil
Source: BMC Complement Med Ther. 2026 Jun 17;26:215. doi: 10.1186/s12906-026-05431-1 (PMC13282879; doi:10.1186/s12906-026-05431-1)
Supplement: Supplementary file 1 — Supplementary Material 1. [file 12906_2026_5431_MOESM1_ESM.pdf]

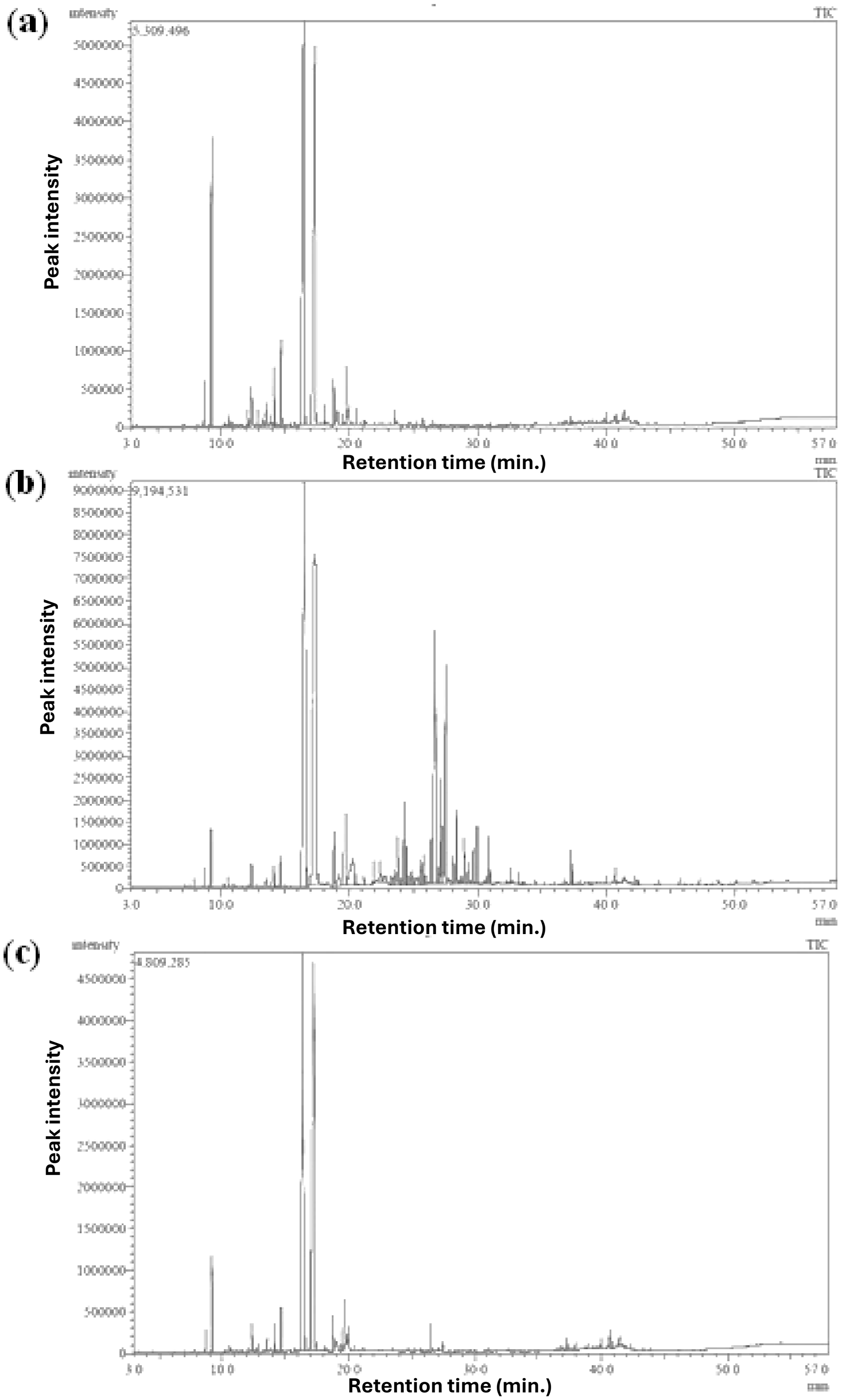

Figure S1. GC-MS chromatograms of *Cymbopogon citratus'*s essential oils from leaves **(a)**, roots **(b)** and stems **(c)**.
