# Supplementary figures and images for: Chemical profile, anti-biofilm and antioxidant activities of Cymbopogon citratus (DC.) Stapf essential oil
Source: BMC Complement Med Ther. 2026 Jun 17;26:215. doi: 10.1186/s12906-026-05431-1 (PMC13282879; doi:10.1186/s12906-026-05431-1)

**(a)**

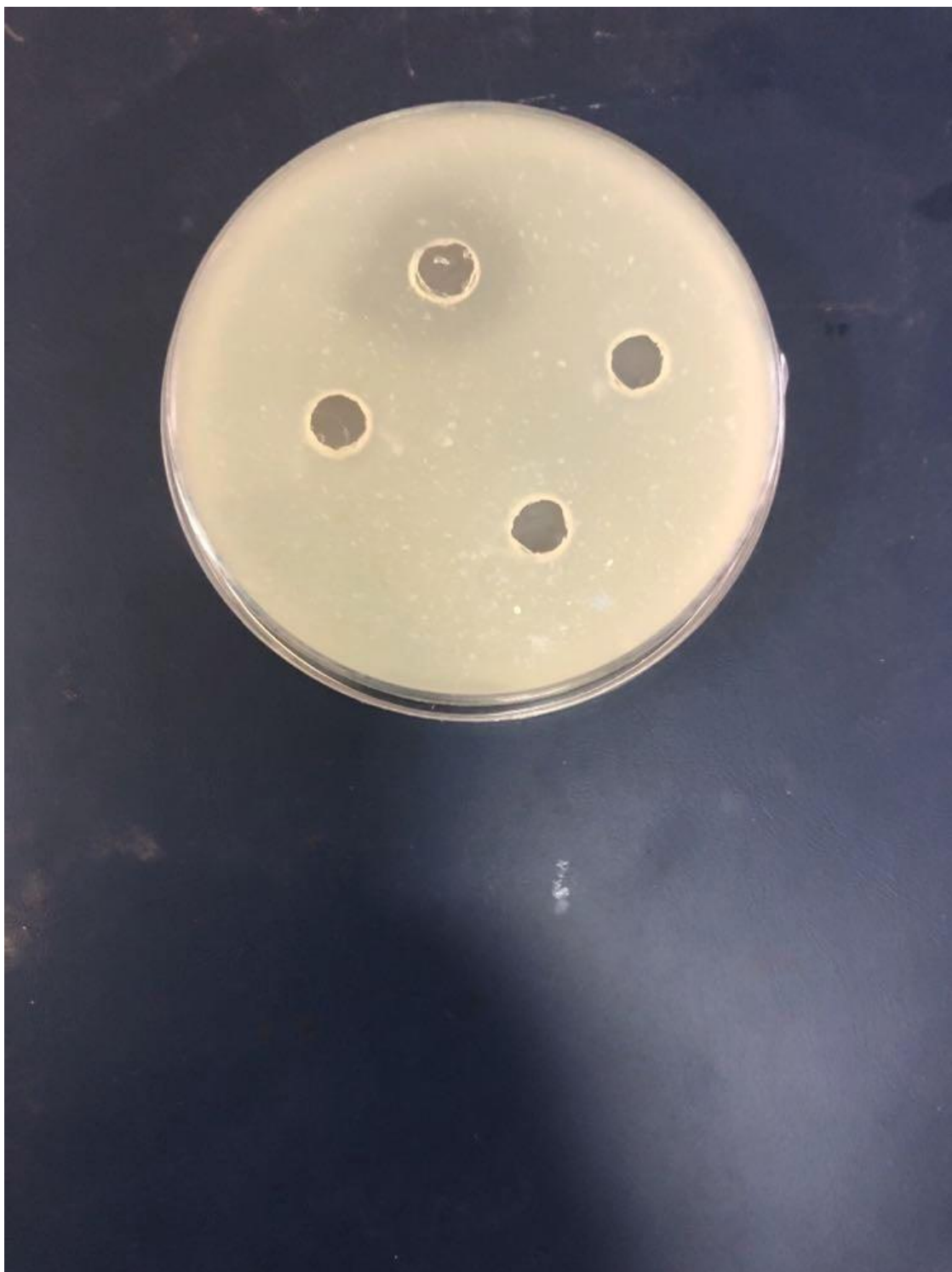

(b)

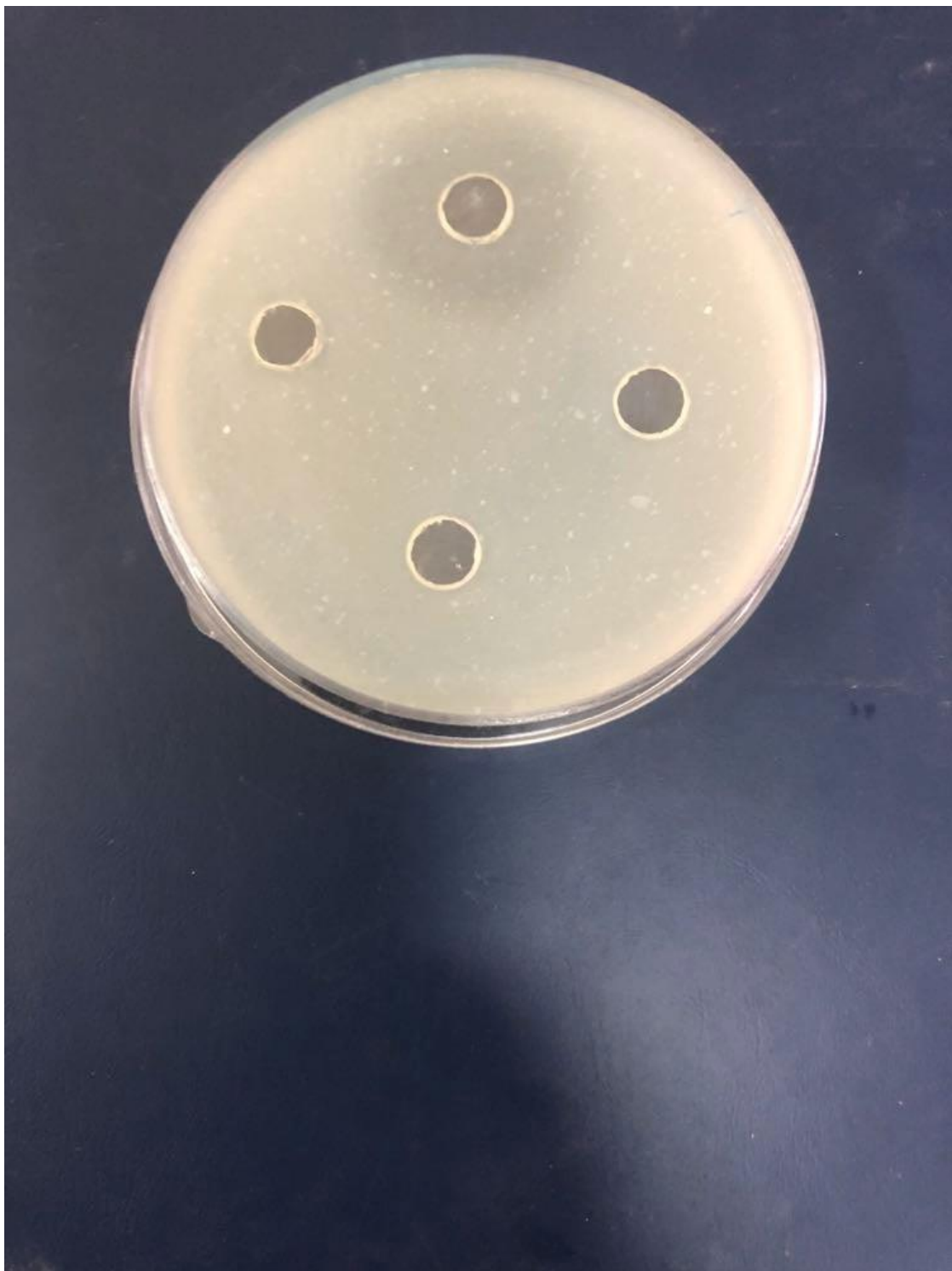

(c)

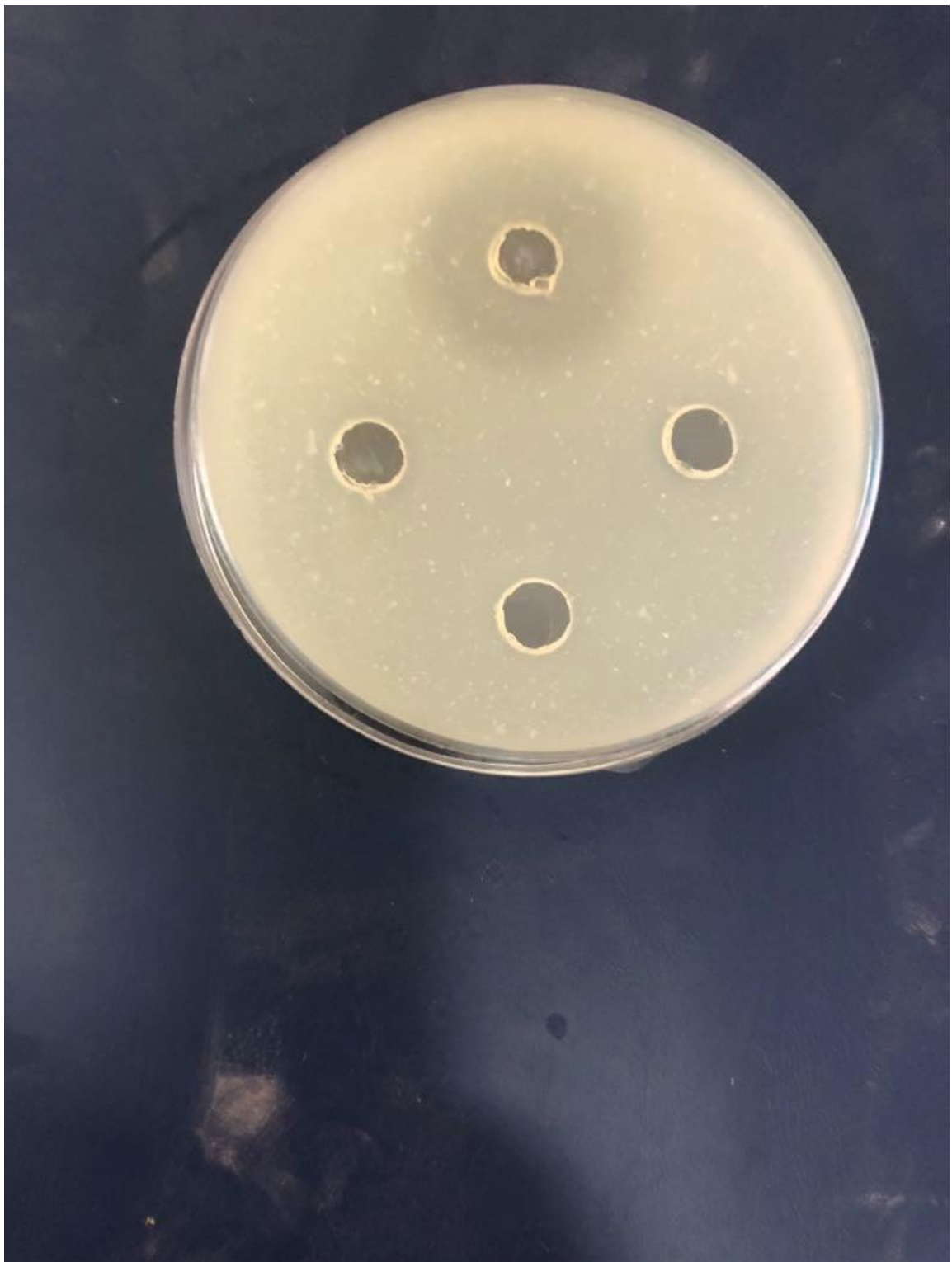

**Figure S2.** Uncropped images of **Fig. 2d**; **(a)** *C. albicans* ATCC 10261, **(b)** *S. aureus* ATCC 6538 and **(c)** *P. aeruginosa* PAO1.

Supplement: Supplementary file 2 — Supplementary Material 2. [file 12906_2026_5431_MOESM2_ESM.pdf]
